# Generational Leaps in Intrapartum Fetal Surveillance

**DOI:** 10.3390/diagnostics15192482

**Published:** 2025-09-28

**Authors:** Lawrence D. Devoe

**Affiliations:** Department of Obstetrics and Gynecology, Medical College of Georgia at Augusta University, 1120 15th Street, Augusta, GA 30907, USA; ldevoe@augusta.edu; Tel.: +1-(706)-721-3556

**Keywords:** electronic fetal monitoring, computer-assisted diagnosis, intelligent systems, risk assessment, artificial intelligence, Fetal Reserve Index, intrapartum surveillance, perinatal outcomes

## Abstract

**Background/Objectives:** Electronic fetal monitoring (EFM) has been used for intrapartum fetal surveillance for over 50 years. Despite numerous trials comparing EFM with standard fetal heart rate (FHR) auscultation, it remains contentious whether continuous monitoring with standard interpretation has reliably improved perinatal outcomes, specifically lower rates of perinatal morbidity and mortality. This review examines previous attempts to improve fetal monitoring and presents future directions for novel intrapartum fetal surveillance systems. **Methods:** We conducted a chronological review of EFM developments, including ancillary methods such as fetal ECG analysis, automated systems for FHR analysis, and artificial intelligence applications. We analyzed the evolution from visual interpretation to intelligent systems and evaluated the performance of various automated monitoring platforms. **Results:** Various ancillary methods developed to improve EFM accuracy for predicting fetal compromise have shown limited success. Only a limited number of studies demonstrated that adding fetal ECG analysis to visual FHR pattern interpretation resulted in better fetal outcomes. Automated systems for FHR analysis have not consistently enhanced intrapartum fetal surveillance. However, novel approaches such as the Fetal Reserve Index (FRI) show promise by incorporating clinical risk factors with traditional FHR patterns to provide higher-level risk assessment and prognosis. **Conclusions:** The shortcomings of visual interpretation of FHR patterns persist despite technological advances. Future intelligent intrapartum surveillance systems must combine conventional fetal monitoring with comprehensive risk assessment that incorporates maternal, fetal, and obstetric factors. The integration of artificial intelligence with contextualized metrics like the FRI represents the most promising direction for improving intrapartum fetal surveillance and clinical outcomes.

## 1. Introduction

Electronic fetal monitoring (EFM) has been used during intrapartum care for more than five decades [1]. While the original goal of EFM used unaided visual interpretation of fetal heart rate (FHR) patterns specifically to reduce perinatal mortality, this goal was subsequently expanded to lower the rates of perinatal morbidity, particularly that associated with fetal/neonatal neurological injury such as hypoxic–ischemic encephalopathy (HIE) and cerebral palsy (CP) [2].

Numerous prior EFM studies have shown that wide variability exists in FHR interpretation, even among experienced clinicians, prompting the need for ongoing attempts to develop more standardized, objective approaches to this modality [3]. Other well-known EFM shortcomings include poor screening test performance, missing data, and the intrusion of FHR signal artefacts.

Here, we explore past efforts to improve EFM as well as to present developments that hold promise for the future, with particular emphasis on intelligent systems that can provide enhanced decision support for clinical management. Throughout this review, the practice patterns, definitions, and recommendations will be based on standards used in the United States of America.

## 2. Previous Attempts to Improve EFM

### 2.1. Standardization Efforts

Three decades ago, the National Institute of Child Health and Human Development (NICHD) assembled an expert panel charged with standardizing the terminology used for visual FHR interpretation [4]. Their deliberations, along with those of a second expert panel convened a decade later, led to an American College of Obstetricians and Gynecologists (ACOG) Practice Bulletin that established a three-category classification system for EFM interpretation [5]. This three-category system was adopted essentially universally in the United States for clinical application while bypassing the usual rigorous studies needed to demonstrate that it actually improved the implementation of EFM or led to better obstetric outcomes.

### 2.2. Ancillary Supportive Technologies

To provide ancillary supportive data to assist in clarifying intrapartum fetal well-being or identify fetal compromise, two noteworthy developments began in the late 1990s.

#### 2.2.1. Fetal Pulse Oximetry

One approach was the design and implementation of fetal pulse oximetry, which was intended to provide continuous recordings that ran concurrently with continuous FHR monitoring. While this system had initial promise [6], a large, randomized trial showed no difference in perinatal outcomes between the group with “open” oximetry signals and the group with “masked” signals [7]. As a consequence, fetal pulse oximetry was abandoned in the United States.

#### 2.2.2. STAN System

A second ancillary system, known as STAN, added real-time analysis of the fetal ECG signal, specifically the ST-segment, to continuous FHR monitoring [8]. This technology had shown promise in European studies, particularly in the reduction of neonatal metabolic acidosis [9]. However, a large NICHD randomized controlled trial failed to show any differences in perinatal outcomes between the study group that had “open source” fetal ST-segment data and the control group in which this information was concealed [10]. Consequently, the STAN monitoring system has not been widely adopted in the United States.

### 2.3. Automated FHR Analysis Systems

An enduring problem that has plagued the use of conventional EFM derives from the observation that very wide ranges of experience and expertise exist among obstetricians and nurses who perform the visual assessment of FHR patterns. Consequently, work began in the late 1980s [11] to develop automated systems capable of intrapartum FHR pattern recognition that can trigger rapid alerts to care providers in a labor and delivery setting.

From these studies, expert systems evolved, like the Oxford Sonicaid (now Sonicaid Fetal Care^®^), which used the Dawes-Redman program to automatically assess FHR tracings but limited its use to antepartum settings [12]. Computerized monitoring programs like OB TraceVue^®^ (now Intellispace Perinatal^®^), Omniview-SisPorto^®^, and PeriWatch Vigilance^®^ [13,14,15] have been used for a number of years to provide intrapartum care in selected obstetrical units around the world.

While all of these systems function as intended for clinical decision support, a very large observational study using Omniview-SisPorto^®^ (which combines computerized FHR analysis and STAN) in a Portuguese tertiary obstetrical unit showed significant decreases in rates of hypoxic–ischemic encephalopathy and cesarean deliveries over a period of 14 years [16]. However, neither this system nor the others mentioned above have been subjected to the most rigorous method of assessment—large randomized controlled trials.

#### The INFANT Study

The largest randomized controlled trial of EFM conducted to date was for INFANT, a decision-support intelligent software platform (K2 Medical Systems) that analyzes FHR signals, color-codes the resulting patterns in terms of their clinical “severity,” and then issues alerts to managing clinicians [17]. The INFANT study enrolled 46,042 pregnant women throughout the UK who were randomized to either (1) a decision-support arm (automated INFANT alerts available) or (2) a control arm with no decision support from the software.

There were two primary outcomes: (1) a composite measure of adverse neonatal outcomes, including deaths and significant morbidity such as HIE; and (2) continuous measures of childhood progress up to age 2. The study found no significant differences in the outcomes of interest between groups for either poor neonatal outcomes or developmental delays. However, these results are questionable as they could have resulted from a possible Hawthorne effect or from a study design in which the same caregivers participated in both study arms, as their continued exposure to the INFANT program may have improved their unaided assessment of FHR recordings.

## 3. Future Directions for Fetal Surveillance Systems

### 3.1. Developmental Stages of Surveillance Systems

**Figure 1:**
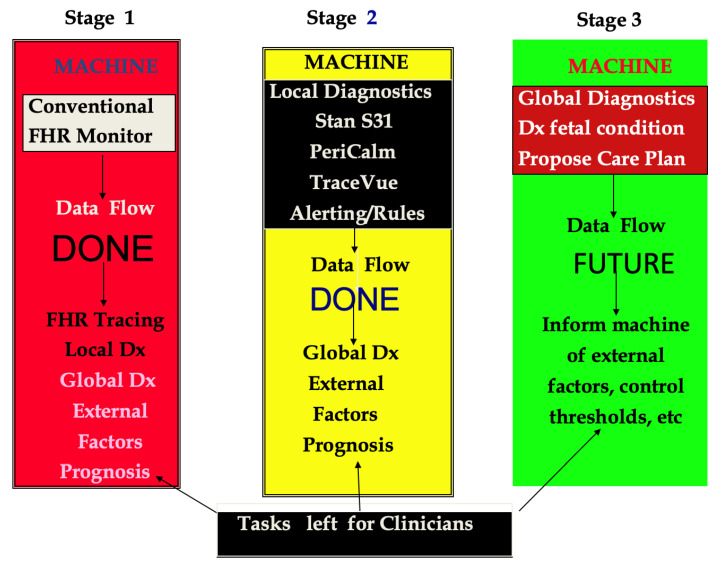
Stages of development of intrapartum surveillance systems.

From an historical perspective, the development of fetal surveillance systems has occurred over the past half-century as improving technological advances have enabled more sophisticated and accurate programs to emerge. Many medical technologies, including those intended for intrapartum care, usually undergo epochs of development, clinical trials and investigations, and then, if these are deemed successful, are deployed into clinical practice. Figure 1 illustrates three discrete developmental stages for fetal surveillance systems over time as the programming for FHR signal recognition becomes more proficient and intelligent decision-support systems eventually appear.

Stage 1: The fetal monitor simply presents the raw, unprocessed FHR and uterine activity data to clinicians who then must visually interpret these data and apply their observations to intrapartum care. This stage marked the introduction of EFM.

**Figure 2:**
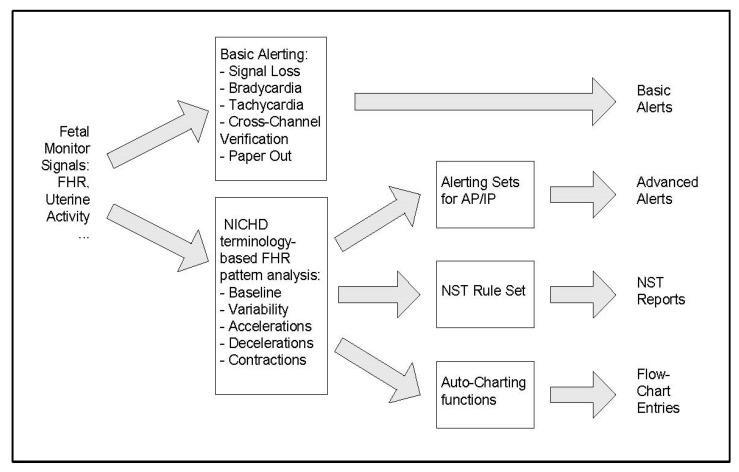
Schematic block diagram of an automated fetal monitor alerting system [After Alonso-Betanzos A, Moret-Bonillo V, Devoe L, Searle J, Banias B, Ramos E. Computerized antenatal assessment: The NST-Expert Project. *Automedica* 1992 14:3–22] [11]. Abbreviations: AP/IP: antepartum/intrapartum; NST: nonstress test.

**Figure 3:**
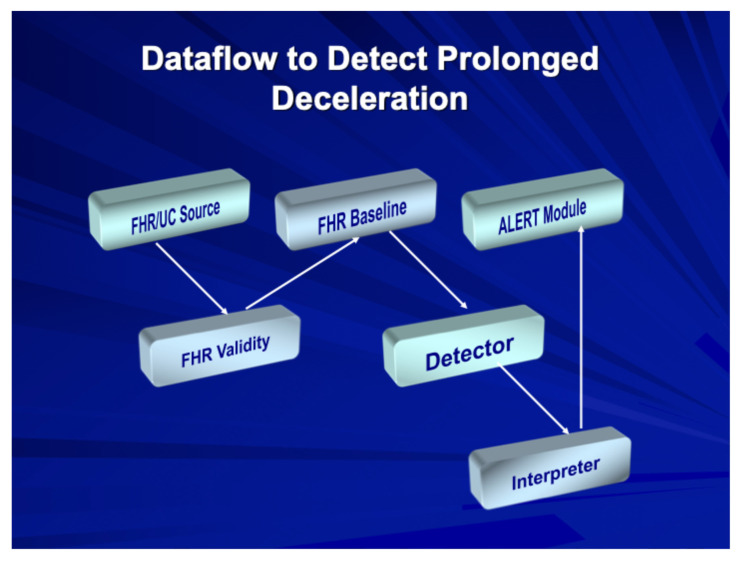
Dataflow during activation of an automated fetal alerting system.

Stage 2: The fetal monitor now provides automated FHR interpretation and/or higher-level alerts for fetal assessment and prognosis. Figure 2 show a typical flow pattern of a rules-based alerting system in which users may opt to engage either basic or more advanced alerts based on features of FHR elements and uterine activity. Figure 3 shows the process of such a system in which the data flow enables validation of the FHR signals and validating FHR baseline and then employing a “detector” for abnormalities, which are routed to an interpreter that then triggers an appropriate alert. Stage 2 represents currently available EFM systems, as shown in Table 1, that are being used for intrapartum surveillance in selected obstetric units (Dx: diagnosis).

Stage 3: The fetal monitor combines additional clinical information about the patient, enabling concurrent perinatal risk assessment that generates alerts and supports management decisions. This stage represents future generation electronic fetal monitors that will translate continuous data and generate accurate risk prediction for adverse intrapartum and neonatal outcomes. Possible features and applications of such systems will be described and discussed in the remainder of this paper.

### 3.2. Intelligent Intrapartum Surveillance Systems

What might future intrapartum surveillance systems of Stage 3 look like? Conventional electronic fetal monitors provided decision-support data that relied solely on FHR pattern interpretation by physicians and nurses. More sophisticated support systems would be required to present specific FHR patterns to large knowledge bases and not only provide user-friendly displays of the conventional EFM elements but also provide prognostic information beyond basic alerting functions.

Future intrapartum surveillance systems must address the fact that the intrauterine environment during active labor is in continual flux and can become chaotic. The ability to predict how a given set of conditions will progress and possibly impact perinatal outcomes currently far exceeds the capability of conventional fetal monitoring systems.

Intrapartum conditions resemble those addressed routinely by the United States’ National Oceanic and Atmospheric Administration (NOAA) to predict natural disasters like hurricanes using satellite networks that send continuous data to intelligent terrestrial computers. The backbone of these systems are hybrid artificial intelligence (AI) programs employing algorithms, expert systems, and neural networks (NNs) to identify and analyze specific patterns associated with severe conditions. In many cases, weather alerts can be issued well ahead of potentially disastrous events and enable life-saving mass evacuations.

### 3.3. Artificial Intelligence Applications

Various efforts have been made to develop computerized solutions for EFM, using any or all of the abovementioned methods to deal with intrapartum environmental variables. A recent narrative review has summarized and evaluated AI programs intended to improve the screening performance of EFM and serves as a baseline for future developments [18]. Table 1 lists automate FHR interpretative programs that have used rule-based expert systems to recognize FHR patterns and classify their occurrence in real time. However, such systems may come up short when they encounter events or conditions for which the rules are lacking or insufficient, which unfortunately is not a rare event.

NNs represent a form of “intelligent” software that, instead of using algorithms or rules, look for associations among variables to predict the probability of outcomes or events of interest. Unlike conventional expert systems, NN solutions improve through “learning” from exposure to increasing numbers of case examples. The reliability and accuracy of such programs depend on their ability to weigh the importance of individual variables through training, testing, and validation. There has been a surge in the reporting of increasingly accurate programs developed for the intrapartum environment, as shown in Table 2 [19,20,21,22,23,24,25,26]. However, few of these intelligent systems have been deployed into clinical practice. Aeberhard et al. [27] have performed a recent scoping review of other AI studies of applications for EFM analysis and prediction of perinatal outcome for those who wish to pursue this topic further.

McCoy et al. [26] developed a deep learning model consisting of neural networks that successfully evaluated a large set of EFM tracings and is the most accurate in terms of predicting the outcomes of interest. Their model focuses on the final 30 min before delivery that predicted ranges of cord blood arterial pH values from <7.05 to <7.20 with high AUROC values and sensitivity. This model was validated with a public-access set of EFM tracings from a previously published Czech database and holds promise for prospective trials.

### 3.4. Risk Assessment Systems

While intrapartum fetal surveillance was intended to provide continuous assessment of fetal condition and issue clinical alerts when the risk for fetal compromise increases, the lack of specificity of FHR patterns has made this an elusive target. Current fetal monitoring systems do not draw upon an extensive clinical database to determine an a priori level of risk present for any given pregnancy upon entry into the labor unit or how this risk may be modified by changes in FHR patterns.

**Figure 4:**
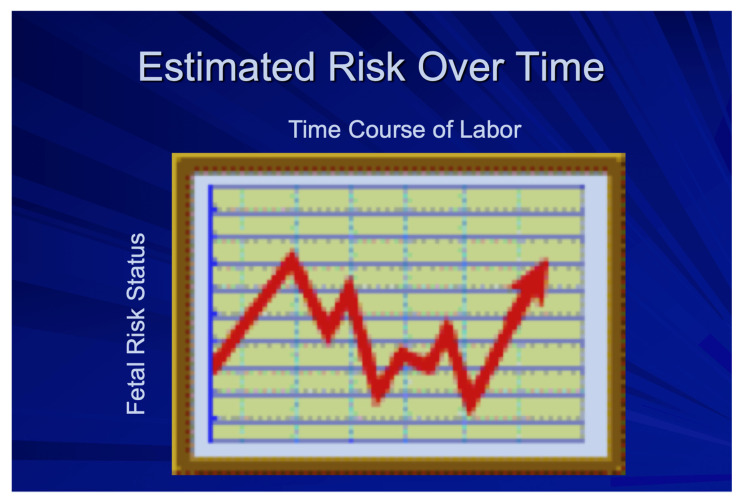
Projection of continuous fetal risk over time (Red Arrow) [After Devoe LDD. Future perspectives in intrapartum fetal surveillance. *Best Pract Res Clin Obstet Gynaecol*. 2016 Jan:30:98–106.doi: 10.1016/j.bpobgyn.2015.06.006. Epub 2015 Jun 25] [28].

Risk levels for fetal compromise may change abruptly and/or continuously during the course of labor. Ideally, the availability of both immediate and progressive alterations in such risks (Figure 4) should be made available to those providing care. Unfortunately, the examples of intelligent systems that have been developed to date (Table 2) may improve the post hoc identification of fetal compromise and/or adverse outcomes, but they do not yield the kind of time-sensitive prospective identification of fetuses whose risks for compromise are changing and for whom intrauterine resuscitation or, in some instances, expedited delivery would be the best intervention.

## 4. The Fetal Reserve Index: A Novel Approach

### 4.1. Conceptual Framework

Fortunately, there are forward-looking solutions to this situation, as described by a series of representative studies listed in Table 3, beginning with the initial presentation of a novel concept for intrapartum assessment—the Fetal Reserve Index (FRI)—a scoring system that leverages the addition of identifiable a priori risk factors (Table 4) to standard EFM features. This construct more closely resembles the practice of medicine in other disciplines: (1) identify clinical risk factors derived from patients’ histories and physical findings and (2) support their most likely outcomes with laboratory tests, in this case, EFM data. The FRI scores were defined in the initial validation studies, using the maternal, obstetrical, and fetal risk factors shown in Table 4 that were combined with additional risk factors stemming from uterine contraction frequency, and FHR features (baseline rate, variability, accelerations and decelerations) in 20-min windows. This results in a continuous measure of intrauterine fetal status that is readily calculable by those providing intrapartum care and updated every 20 min. The original scores were derived empirically and then retrospectively evaluated on well-documented cases [29].

The FRI has been developed as a proof-of-concept approach developed exclusively on US populations. Its development has been summarized in a 2023 review article [29]. We are in the process of converting the platform to a machine learning approach that will assign weights to the FRI parameters and refine the performance of the FRI for possible use in other populations. Definitions of what is normal or abnormal for fetal growth or umbilical Doppler studies are also based on US standards. We have performed one retrospective study that demonstrates the potential for the early determination of possible fetal compromise to provide timely intervention and prevent adverse outcomes [33]. This limited prospective study applied the FRI scores and resulting colorimetric zones to institute timely intrauterine resuscitation and safely lower rates of emergency operative deliveries [EODs] without compromising perinatal outcomes. Clearly, larger prospective studies would be needed. In our current project to develop an AI platform for the FRI, preliminary and unpublished data show that a window for accurately identifying risk for adverse outcomes usually occurs at least three hours before delivery which, if confirmed by prospective studies, would enable enough time for appropriate obstetric interventions required to confirm these observations, and we plan on conducting such studies in the near future.

### 4.2. Initial Validation Studies

The initial FRI study [28] assessed the ability of the FRI to retrospectively examine a population of 50 CP cases and 200 controls who all entered labor in normal condition. The FRI predictions were compared with those associated with ACOG Category III fetal tracings [5] and the criteria listed in an ACOG monograph for the neonatal identification of CP [30].

FRI scores were assigned by combining FHR features (baseline rate, variability, accelerations, and decelerations) with uterine contraction frequencies and fetal, maternal, and obstetric risk factors. Scores ranged from 1.0 (no risk factors present) to <0.25 (at least six risk factors present). The scores subsequently assign fetuses to colorimetric risk zones of green (low risk, 1.0–0.5), yellow (moderate risk, <0.5–0.25), and red (high risk, <0.25). When risk factors are not known, they will not be considered in determining the FRI score or colorimetric risk zone.

**Figure 5:**
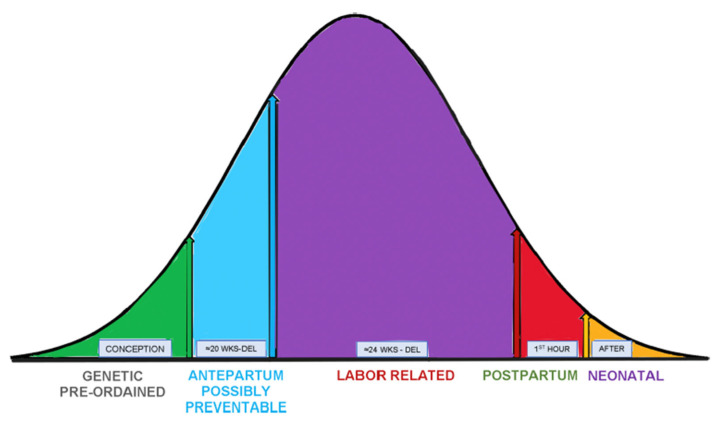
Etiology and ontogeny of cerebral palsy. Distribution curve of CP cases. [After Evans MI, Britt DW, Devoe LD. Etiology and ontogeny of cerebral palsy: Implications for practice and research. *Reprod Sci* (doi.org/10.1007/s43032-023-01422-6 Epub 2023 Dec 22)] [39].

Using this approach, the FRI scoring system identified 100% of CP cases, while the ACOG Category III FHR tracings and ACOG CP monograph identified 44% and 30% of cases, respectively. The etiology of CP covers a spectrum of both well-established and hypothetical causes [38] (Figure 5), and the ability to identify contributing intrapartum conditions that could be averted was a very promising avenue for the FRI to pursue. This is illustrated in a follow-up case–control series in which 420 patients were admitted with normal FHR tracings, of which 60 developed CP [39]. Compared to CP cases, all of which reached and remained in the red zone for at least two hours, control patients who reached the red zone were delivered more expeditiously and often had prompt management including IR maneuvers, which improved the FRI and lowered the risk of emergent operative deliveries (EODs), i.e., cesarean and operative vaginal deliveries, even for cases with normal outcomes.

### 4.3. Clinical Applications

The potential of the FRI scoring system to achieve better labor and delivery management has been investigated in several studies. In one study of 300 patients with normal neonatal outcomes reviewed for the outcome of emergent operative delivery (EOD) [40], FRI scores were much lower in EOD cases that also spent longer durations in the red zone.

A subsequent study [31] examined 1402 term singleton pregnancies with normal outcomes and found that if patients entered the red zone and remained there for more than one hour, intrauterine resuscitation (IR) in the first stage of labor lowered the probability for EOD from 93% to 15%.

A prospective observational study of 400 control patients and 400 patients assessed with the FRI, all with normal outcomes, showed similar incidences of red zone tracings (25%) [33]. When the FRI score was taken into consideration and IR was performed, EODs decreased from 17.3% to 4.0%.

While IR remains a cornerstone of intrapartum management, particularly in the setting of induced or augmented labor, the ability to establish a safe threshold for uterine contraction frequency (UCF) and to assess the impact of IR on fetal condition have long been contentious issues. Regarding the first topic, the current ACOG criteria have set the normal upper limit for mean uterine contraction frequency at 5 contractions per 10 min, averaged over a 30-min window [5]. Excessive uterine activity clearly can be deleterious to fetal intrapartum well-being as it decreases the intervals for placental reperfusion and fetal oxygenation. A study of 475 closely monitored term patients was subdivided into two groups based on UCF thresholds exceeding either four or five contractions per 10 min during the last hour of labor [32]. Measured outcomes included cord blood pH, base excess, Apgar scores at 1 min, neonatal heart rate at 16 min after birth, and the proportion of births that did not result from normal spontaneous vaginal deliveries. Table 5 shows a comparison of the two UCF thresholds associated with the above outcomes, making it clear that a UCF threshold of four UCs per 10 min provided superior predictive performance.

The above set of observations prompted a focused review of a case series of 118 patients receiving Pitocin for either the induction or augmentation of labor [34]. Recognizing that no standardized definitions exist for what constitutes IR or how its impact is assessed, two measures of relative IR effectiveness were determined over a two-hour time frame after Pitocin was first initiated. Levels of fetal risk severity at the time of IR initiation were then examined to determine how IR effectiveness was affected and clinical decisions were made. Differences in the IR practices that were observed during this retrospective survey dealt with timing of IR and choice of Pitocin management, which were undertaken without the benefit of an a priori FRI score or colorimetric risk zone assignment. As all IR “maneuvers” included were accompanied with changing maternal position and administering supplemental oxygen, these latter IR components were not considered as variables. Three scenarios of Pitocin management were constructed: (1) no reduction in Pitocin infusion rates; (2) decreased Pitocin infusion; and (3) Pitocin discontinued. FRI scores were determined over six consecutive 20 min windows and IR response was characterized as (1) improvement (higher FRI score relative to start of IR) or (2) stabilization (no further decrease in FRI score relative to its value at the start of IR). The first and/or only initiation of IR led to improvement in 71% of cases and stabilization in 78% of cases. IR failures accounted for the remaining 22% of cases as the FRI score in the sixth 20 min period was lower than the FRI score at the time of IR initiation. There were no differences in percent improvement or stabilization of FRI scores as a function of the level of risk at the time of IR initiation. That said, using calculated FRI scores to assess IR response could be an objective framework to measure IR effectiveness, as applied to future studies.

### 4.4. Correlation with Acid–Base Status

**Figure 6:**
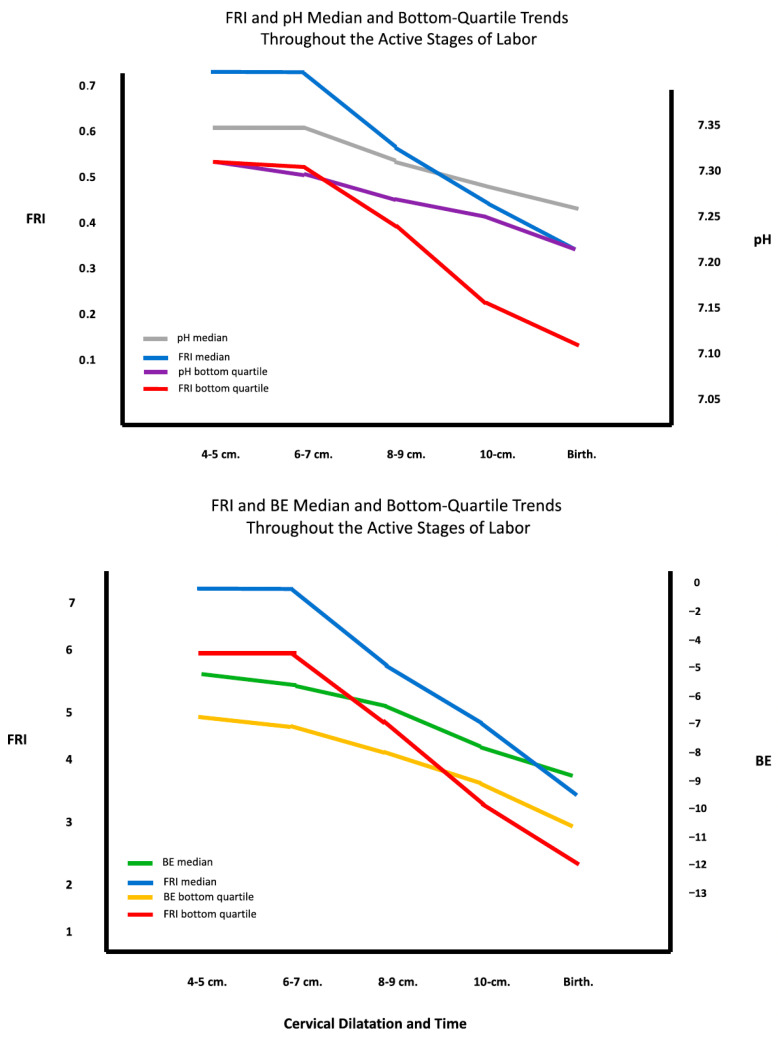
Correlations of FRI with pH [**top**] and base excess [**bottom**] medians over time. After Evans MI, Britt DW, Evans SM, Devoe LD. Improving the interpretation of electronic fetal monitoring: the fetal reserve index. *Am J Obstet Gynecol.* 2023 May; 228(5S): S1129–S1143. doi: 10.1016/j.ajog.2022.11.1275 [29].

A further benefit from studying such well-documented populations as those described above was the ability to correlate acid–base balances both during labor, provided by fetal scalp blood sampling [37], and at delivery by umbilical cord blood analysis [36]. In both instances, there was good correlation of pH and base excess trends over time with concurrent FRI scores (Figure 6). These are important findings since fetal scalp blood sampling is no longer routinely performed, and the FRI could be considered a potential continuous surrogate measure for such assays during labor. We have also demonstrated that the FRI outperformed the ACOG 3-Category system in predicting fetal cord blood pH and Base Excess [41].

Any new approach to clinical care like FRI often elicits criticism and resistance to its acceptance, as was illustrated in two recent articles that were either dismissive [42] or simply misinformed [43]. The first publication [42] appeared at a time when the FRI had received its first four publications that represented our initial attempts at “proof of concept” studies. Its author contends that this “system has never been validated, there has been lack of standard criteria for diagnosing cases of HIE, lack of appropriate controls, and multiple obvious sources of bias.” As borne out by our studies, these statements are simply untrue. Subsequently, there have been 15 additional peer-reviewed publications that support our initial investigations and continue to strengthen the FRI concept as a valid approach that could greatly improve the performance of EFM. The second publication [42] considered the FRI to be “junk science.” It attempted to support this contention with several patently false statements, including allegations that all of the original FRI study patients were derived from medical malpractice cases and that FRI studies could predict the exact timing of intrapartum fetal injury. Our development of the FRI approach has been described in this paper and future avenues for intrapartum monitoring systems and their outcomes are briefly described in the following section, which should effectively put such off-base criticisms to rest.

## 5. Future Horizons for Improving Intrapartum Monitoring Systems

### 5.1. Limitations of Current Approaches

Innovations for intrapartum fetal monitoring have had mixed results due, in no small part, to the unpredictability of the intrauterine environment in which fetal oxygenation is continually challenged by repetitive uterine contractions. As previously noted, attempts to incorporate fetal scalp blood pH, fetal scalp oximetry, or ECG analysis into clinical management have been abandoned by the US obstetrical community. There are a number of new attempts to try again to use fetal EEGs, pulse oximetry, and AI-guided systems. It is certainly possible, but nowhere proven, that such attempts will have much better outcomes this time than previously. Without incorporating the larger ecosystem of fetal reserve (i.e., adding the risk factors), the asymptote of success will likely still be suboptimal to the use of a more contextualized metric.

### 5.2. AI-Enhanced FRI Development

Given the rapid advances in AI technology in medicine in general and obstetrics specifically, the previous section provided examples of such developments focused on intrapartum fetal monitoring. However, simply improving the accuracy of FHR pattern analysis is only one part of any future intelligent solutions. Achieving the successful future development of an AI-enhanced FRI model will require significantly large population datasets since the occurrence of the adverse outcomes of interest is relatively rare. As noted, and unlike the other approaches previously described, the FRI adds valuable context in real time to EFM and functions much like a clinician would in making patient assessments to aid ongoing clinical management. The next step for FRI development is to provide an AI platform in which the following steps would take place:

1. **Automated FHR Analysis**: Enable extraction of the four features (baseline FHR, variability, accelerations, and decelerations) and uterine activity quantification to determine uterine contraction frequencies.
2. **Electronic Health Record Integration**: Incorporation of maternal, fetal, and obstetrical risk factors directly from electronic health records.
3. **Machine Learning**: Weighting of risk factors with machine learning to create AI programs, most likely in the form of neural networks.
4. **Real-time Scoring**: Automated FRI score generation updated every 10 to 20 min, generating clinical alerts when necessary (e.g., fetal status changes as colorimetric zones transition from green to yellow or from yellow to red).
5. **Intervention Tracking**: When IR is being performed, associated FRI scores would track its impact on fetal status at 20 min intervals.
6. **Cloud Accessibility**: FRI system output would be accessible in secure cloud servers that could notify care providers at any location.
7. **Remote Monitoring**: The FRI system would provide the backbone for remote fetal monitoring at home with the capability of notifying providers of changes in status.
8. **Postnatal Surveillance**: Postnatal fetal surveillance using FRI principles for continuous FHR monitoring and transcutaneous pulse oximetry for the first hour of neonatal life. The need for such surveillance is suggested by patterns of abnormal neonatal physiologic adaptation for compromised infants that were observed in one of our earlier studies [36].
9. **Resource-Adapted Versions**: Versions of the FRI system would be developed for low-resource settings.

It is also likely that other modalities like fetal electroencephalography and oximetry using transabdominal probes will continue to be explored as alternatives or additions to conventional EFM, as has been achieved to varying degrees for more than two decades [44,45]. While signal capture and rendering technologies have continued to improve, most of such studies have been conducted in laboratory animals, and experiments in human subjects remain limited. Regarding the latter modality, there is a new system for fetal pulse oximetry being developed by Raydiant Oximetry (https://radiantoximetry.com) that consists of a transabdominal set of sensors intended to detect and track fetal arterial blood oxygen saturation. This system is currently engaged in an early feasibility study.

### 5.3. Regulatory and Implementation Considerations

What must be understood from the beginning is that novel systems, particularly those relying on AI programs, require extensive training, testing, and validation, which are often very costly. In the US, such systems must undergo review by the Federal Drug Administration (FDA) as devices [46]. The first step involves a pilot study demonstrating proof of concept, i.e., the system works as described, and that it satisfies patient safety requirements. Should FDA approval then be granted, there would be a requirement that the system performance be monitored once it is introduced into practice to ensure that it does not contribute to unexpected or adverse outcomes. However, to convince potential clinical users that the novel system provides a significant management improvement over conventional monitoring systems, a sufficiently powered randomized controlled trial would be essential. Assuming the RCT favors the novel system, its parent company would need to roll out a marketing program and provide clinical education courses for its new clients. Again, assuming that sufficient numbers of early adopters appear, wider acceptance by the obstetric community would gradually follow over a period of time that might require years. History does not suggest the rapid adoption of almost anything novel. Most new practices have taken well over a decade to gain acceptance.

### 5.4. Medico-Legal Considerations

A final consideration for the development and rollout of novel intrapartum monitoring systems involves medico-legal liability implications for users and vendors [47]. Fetal monitoring has well-known risks, and companies willing to accept these risks must be covered for potential litigation cases. Any time new products are developed and deployed, the bottom line must be the company’s willingness to invest in them without the guarantee of profitability.

## 6. Conclusions

This review has highlighted the obvious limitations of conventional EFM and current efforts to improve this modality. While automated systems for FHR feature recognition represent a step forward, without ancillary risk assessment, they remain limited in their ability to provide a real-time and accurate assessment of fetal condition.

Pathways to improve intrapartum fetal surveillance are clear and this monograph has highlighted a few of them. The ultimate solution for smarter intrapartum surveillance systems will inevitably combine conventional fetal monitors with intelligent programs to capture and assess, in real time, the large amount of data that a single labor can generate. With the advent of a more comprehensive approach like the FRI, this solution is now closer than ever.

The integration of artificial intelligence with comprehensive risk assessment represents the most promising direction for advancing intrapartum fetal surveillance. Future systems must move beyond simple pattern recognition to incorporate the full clinical context, providing healthcare providers with actionable intelligence that can improve both maternal and neonatal outcomes while reducing unnecessary interventions.

## Figures and Tables

**Table 1:** Automated systems for FHR assessment.

Authors	System Name	Design	Data Analyzed	Year
Dawes GS et al. [12]	Oxford Sonicaid	Rule-based	Antepartum FHR	1996
Alonzo-Betanzos A et al. [11]	NST-Expert	Rule-based	Antepartum FHR	1992
Keith RDF et al. [13]	INFANT	RB + AI *	Intrapartum FHR	1994
Bernades J et al. [14]	SIS-Porto	Rule-based	Intrapartum FHR	2000
Hamilton et al. [15]	Peri-Calm	RB + AI	Intrapartum FHR	2010
Amer-Wahlin et al. [8]	STAN	Rule-based	IP FHR + fetal ECG	2014

* Artificial intelligence (neural networks).

**Table 2:** Representative studies of AI applications for EFM analysis and perinatal outcome prediction.

Reference	Study Design	Study Population	AI Model	Accuracy Metric	Category	Benefits
Georgieva et al., 2013 [19]	Retrospective	124 patients: training set 252 patients: testing set 7568 patients: validation set	NN	PPV: 84.8% NPV: 81.5% Sensitivity: 80% Specificity: 85%	EFM	Highly accurate model for predicting adverse outcomes
McCoy et al., 2025 [26]	Retrospective	10,182 patients, time-stamped matched with cord blood gases with pH values	NN	AUROC: 0.89	EFM Clinical Care	Correlation of pH values < 7.05 and BE of <−10 meq/L With EFM patterns
Lee et al., 2023 [25]	Retrospective	5249 cardiotocography traces	NN	Harmonic means 0.94–0.98	Analyze EFM	Efficient model for fetal status classification in real time
Zhang et al., 2024 [24]	Retrospective	2542 CTG records	SVM NN	AUROC: 0.95	Analyze EFM	Effectively assists obstetricians in classifying CTG i
Daydulo et al., 2022 [23]	Retrospective	552 recording	NN	AUROC: 0.78	EFM Clinical Care	Can be used as a decision-making aid system for obstetricians
Ogasawara et al., 2021 [22]	Retrospective	5406 deliveries	NN	AUROC: 0.73	EFM Clinical Care	Reliably predicted infant outcomes from 30 min of CTG just before delivery
Park et al., 2022 [21]	Retrospective	1456 recordings	NN, LGBM	AUROC: 0.73	Analyze EFM	Precise FHR signal classifying as a screening tool to monitor fetal status
Al-Yousif et al., 2022 [20]	Retrospective	150 CTG signals	Fuzzy logic	Kappa scores ranging from 0.607 to 0.926	Analyze EFM	Good prediction of the visual classification results by three expert panelists

Abbreviations: AUROC: area under a receiver-operating characteristic curve; NN: neural networks; SVM: support vector machine; LGBM: light gradient-boosting machine.

**Table 3:** Representative FRI studies.

Representative FRI Studies	Population	Study Type	Objective	Results
Eden RD, Evans MI, Evans SM, Schifrin BS. The Fetal Reserve Index: Re-engineering the interpretation and responses to fetal heart rate patterns *Fetal Diagn Ther*. 2018; 43(2):90–104 [30]	50 CP cases 200 controls All cases normal on admission	Retrospective	Compare FRI to ACOG Categories and CP Monograph to identify CP	For CP cases FRI scores identified 100%, ACOG Category III identified 44%, ACOG CP Monograph criteria found 30%.
Eden RD, Evans MI, Evans SM, Schifrin BS. Re-engineering electronic fetal monitoring interpretation: Using the Fetal Reserve Index to anticipate the need for emergent operative delivery. *Reprod Sci*. 2018 Apr; 25(4): 487–97 [31]	300 cases with normal neonatal outcomes	Retrospective	Compare FRI scores and zones for EOD cases with non-EOD cases	EOD cases << FRI scores than non-EOD cases. “Red zone” more often and longer for EOD cases with sensitivity of 92%, PPV of 64%, and false positive rate of 10%.
Eden RD, Evans MI, Britt DW, Evans SM, Schifrin BS. Safely lowering the emergency cesarean and operative vaginal delivery rates using the Fetal Reserve Index. *J Matern Fetal Med* 2020 May;33(9):1473–1479 [32]	400 control; 400 using FRI All normal outcomes	Prospective	Predict risk of EOD if FRI principles used in management	Comparable incidence of red zone tracings (25%). IR in 1st group (20%), in 2nd group (47%). EODs reduced from 17.3% to 4%.
Britt DW, Evans MI, Schifrin BS, Eden RD. Refining the prediction and prevention of emergency operative deliveries with the Fetal Reserve Index. *Fetal Diag Ther* 2019; 46:159–165 [33]	1402 term singletons in labor with normal outcomes	Retrospective	Predict EOD risk in FRI Red zone ≥ 1 h and if IR performed	Reaching Red zone early and remaining > 1 h increases EOD probability. When these risk factors are paired with IR in Stage 1, EOD probability is reduced from 0.93 to 0.15.
Evans MI, Britt DW, Worth J, Mussali G, Evans SM, Devoe LD. Uterine contraction frequency in the last hour of labor: how many contractions are too many? *J Matern Fetal Neonatal Med* 2022 Dec; 35 (25):8698–8705 [34]	475 patients monitored in labor and neonatally	Retrospective	Evaluate CB BE, and pH; 1′ Apgar, non-NSVDs, NHR@16′ postnatal	UCF > 4: higher sensitivity to detect decreased Apgar-1′ and 5′, NHR above 160 bpm, higher BE, and non-NSVD than UCF > 5, earlier fetal compromise detected.
Evans MI, Britt DW, Eden RD, Evans SM, Schifrin BS. Earlier and improved screening for impending fetal compromise. *J Matern Fetal & Neonatal Med* 2022 Dec, 35 (15): 2895–2903 [35]	475 high-risk patients monitored in labor and neonatally	Retrospective	Assess FRI score as a proxy for fetal pH and BE values from fetal scalp sampling (FSS)	FSS-obtained pH and BE worsens during 1st stage of labor. Trajectory of FRI provides reasonable approximation of FSSpHBE trajectory to enable earlier intervention as needed.
Eden RD, Evans MI, Britt DW, Evans SM, Gallagher P, Schifrin BS. Combined prenatal and postnatal prediction of early neonatal compromise risk. *J Maternal-Fetal & Neonatal Medicine* 2021, 34 (18); 2996–3007 [36]	251 high-risk singleton term pregnancies	Retrospective	Last FRI score to immediate NHR pattern and umbilical/NN acid-base balance	FRI successfully predicted neonates with suboptimal adaptation and need for additional support.
Devoe LD, Britt DW, Macedonia CR, Worth JM, Mussalli GM, Mondestin-Sorrentino M, Evans MI: Reconceptualizing intrauterine resuscitation and its short-term impact. *Diagnostics* 2025; 15: 255. doi.org/10.3390/diagnostics15030255 [37]	118 patients receiving Pitocin to induce or augment labor and who had IR	Retrospective	Derived 2 measures of IR effectiveness: (1) Improvement (2) Stabilization based on FRI score change	71% improved; 78% stabilized with IR by FRI score changes. However, wide variation in clinician practices for using IR were noted and did not necessarily correlate with FRI-calculated fetal risk.

Abbreviations: CP: cerebral palsy; EOD: emergent operative deliveries; NSVD: normal spontaneous vaginal delivery; IR: intrauterine resuscitation; UCF: uterine contraction frequency; CB: cord blood; BE: base excess; NHR: neonatal heart rate.

**Table 4:** Fetal Reserve Index risk factors.

Maternal Risk Factors	Obstetric Risk Factors	Fetal Risk Factors
Decreased cardiac output/vascular perfusion of the placenta - Cardiac disease with risk of reduced cardiac output in pregnancy - Hypertension (chronic, pregnancy-induced - SLE	IUGR/Macrosomia.	Abnormal Dopplers/BPP
Oxygen carrying capacity - Pulmonary disorders (e.g., asthma) - Anemia and hemoglobinopathy	Oligohydramnios	Genetic Disorders
Infection (chronic and acute)	Polyhydramnios	Fetal arrhythmia
Chronic debilitating disease	Bleeding and abruption	Meconium passage
Malabsorption/poor weight gain	Previous cesarean section	Chorioamnionitis
Endocrine—diabetes and thyroid disorders	Placental and umbilical cord anomalies	Second stage of labor-pushing
Advanced maternal age	Rupture of membranes PPROM, SROM, AROM	Amnioinfusion
Drug abuse, addiction and smoking	Dystocia (protraction, arrest labor disorders)	Discontinuation of Pitocin due to fetal intolerance
Obesity—BMI > 35	Malpresentation	Conversion pattern (acute prolonged tachycardia > 170 bpm)
Short stature ≤ 5′2″		Ominous overshoots
		Bradycardia (<100 bpm)
		Missing important data in labor (e.g., lack of EFM in second stage)

After Eden RD, Evans MI, Evans SM, Schifrin BS. The fetal reserve index: Re-engineering the interpretation and responses to fetal heart rate patterns. *Fetal Diagn Ther* 2018; 43:90–104. Doi: 10.1159/000475927 [30].

**Table 5:** Comparison of UCF > 4 and UCF > 5 with respect to metrics.

Uterine Contraction Frequency (No./Min.)	Apgar1′ * Sensitivity (Specificity) [PPV] PLR	Apgar5′ * Sensitivity (Specificity) [PPV] PLR	NHR 16 * Sensitivity (Specificity) [PPV] PLR	Non-NSVD * Sensitivity (Specificity) [PPV] PLR	BEcg * Sensitivity (Specificity) [PPV] PLR	pHcg * Sensitivity (Specificity) [PPV] PLR
UCF > 4 **	79% (28%) [17%] 1.10	74% (28%) [27%] 0.97	81% (31%) [52%] 1.19	78% (31%) [41%] 1.12	82% (29%) [16%] 1.16	84% (29%) [15%] 1.18
UCF > 5 **	38% (67%) [18%] 1.17	34% (67%) [8%] 1.02	38% (68%) [52%] 1.17	37% (68%) [42%] 1.17	37% (28%) [16%] 1.12	32% (66%) [13%] 1.02

* Apgar1′ is cut at 5. and * Apgar5′ is cut at 5. NHR16 (Neonatal Heart Rate) is defined as not decreasing to 160 bpm at 16 min after birth. * NSVD (normal spontaneous vaginal delivery) is binary by definition. * BEcg (Base Excess) is cut at −12 mM/L and * pHcg is cut at 7.18. ** UCF > 4 is a dichotomy defined as any 10-min periods in the last hour with more than 4 contractions. UCF > 5 is a dichotomy defined as any 10-min periods in the last hour with more than 5 contractions. After Evans MI, Britt DW, Worth J, Evans SM, Mussalli G, Devoe LD: Uterine Contraction Frequency in the last hour of labor: how many contractions are too many? *J Matern Fetal Neonatal Med* 2022 Dec; 35(25): 8698–8705 [34].

## Data Availability

No new data were created or analyzed in this study. Data sharing is not applicable to this article.
